# COVID-19 Vaccination in Fragile Patients: Current Evidence and an Harmonized Transdisease Trial

**DOI:** 10.3389/fimmu.2021.704110

**Published:** 2021-08-10

**Authors:** Chiara Agrati, Serena Di Cosimo, Daniela Fenoglio, Giovanni Apolone, Fabio Ciceri, Gennaro Ciliberto, Fausto Baldanti, Massimo Costantini, Diana Giannarelli, Giuseppe Ippolito, Franco Locatelli, Alberto Mantovani, Aldo Morrone, Fabrizio Tagliavini, Antonio Uccelli, Pier Luigi Zinzani, Nicola Silvestris, Maria Rescigno

**Affiliations:** ^1^Cellular Immunology Laboratory, Department of Epidemiology, Preclinical Research and Advanced Diagnostic, National Institute for Infectious Diseases (INMI) Lazzaro Spallanzani - IRCCS, Roma, Italy; ^2^Biomarkers Unit, Department of Applied Research and Technological Development, Fondazione IRCCS Istituto Nazionale dei Tumori, Milano, Italy; ^3^Department of Internal Medicine and Centre of Excellence for Biomedical Research, University of Genoa, Genoa, Italy; ^4^Biotherapies Unit, IRCCS Ospedale Policlinico San Martino, Genoa, Italy; ^5^Scientific Directorate, Fondazione IRCCS Istituto Nazionale dei Tumori, Milano, Italy; ^6^Scientific Directorate, IRCSS San Raffaele Scientific Institute, Milano, Italy; ^7^IRCCS Regina Elena National Cancer Institute, Istituti Fisioterapici Ospitalieri (IFO), Rome, Italy; ^8^Molecular Virology Unit, Fondazione IRCCS Policlinico San Matteo; Dpt. of Clinical, Surgical, Diagnostics and Pediatric Sciences, University of Pavia, Pavia, Italy; ^9^Scientific Directorate, Azienda Unita Sanitaria Locale (USL)-IRCCS Reggio Emilia, Reggio Emilia, Italy; ^10^Biostatistical Unit, Istituto Nazionale Tumori Regina Elena IRCCS - IFO, Rome, Italy; ^11^Scientific Directorate, INMI Lazzaro Spallanzani - IRCCS, Roma, Italy; ^12^Department of Pediatric Hematology and Oncology, IRCCS Ospedale Pediatrico Bambino Gesù, Department of Gynecology-Obstetrics and Pediatrics, University “La Sapienza”, Roma, Italy; ^13^Scientific Directorate, IRCCS Humanitas Clinical and Research Center, Rozzano, Italy; ^14^Department of Biomedical Sciences, Humanitas University, Milan, Italy; ^15^William Harvey Research Institute, Queen Mary University, London, United Kingdom; ^16^Scientific Directorate, San Gallicano Dermatological Institute IRCCS, Rome, Italy; ^17^Scientific Directorate, Fondazione IRCCS Istituto Neurologico Carlo Besta, Milan, Italy; ^18^Department of Neuroscience, Rehabilitation, Ophthalmology, Genetics, Maternal and Child Health and Center of Excellence for Biomedical Research (CEBR), University of Genoa, Genoa, Italy; ^19^IRCCS Ospedale Policlinico San Martino, Genoa, Italy; ^20^IRCCS Azienda Ospedaliero-Universitaria di Bologna Istituto di Ematologia “Seràgnoli”, Bologna, Italy; ^21^Dipartimento di Medicina Specialistica, Diagnostica e Sperimentale Università di Bologna, Bologna, Italy; ^22^Medical Oncology Department, IRCCS Istituto Tumori “Giovanni Paolo II”, Bari, Italy; ^23^Department of Biomedical Sciences and Human Oncology, University of Bari “Aldo Moro”, Bari, Italy; ^24^Mucosal Immunology and Microbiota Unit, IRCCS Humanitas Clinical and Research Center, Milano, Italy

**Keywords:** vaccine, cancer, immunocompromised, COVID-19, myasthenia, SARS-CoV-2

## Abstract

Patients diagnosed with malignancy, neurological and immunological disorders, *i.e.*, fragile patients, have been excluded from COVID-19 vaccine trials. However, this population may present immune response abnormalities, and relative reduced vaccine responsiveness. Here we review the limited current evidence on the immune responses to vaccination of patients with different underlying diseases. To address open questions we present the VAX4FRAIL study aimed at assessing immune responses to vaccination in a large transdisease cohort of patients with cancer, neurological and rheumatological diseases.

## Introduction

The currently emergency authorized mRNA- COVID-19 vaccines - Moderna (1) and Pfizer BioNTech (2) have been evaluated in clinical trials that excluded, in accordance with the current regulations, immunocompromised subjects, and restricted participation to patients with stable underlying conditions, *i.e.*, stable HIV infection. As a consequence of the relative lack of information, the concern of a potentially reduced efficacy of the vaccines in subjects with altered immunocompetence has arisen. In addition, the characterization of a population broadly defined as “with altered immunocompetence” in relation to the vaccination is yet to be determined. Immunosuppressive conditions, as those induced by immunological disorders and medications, may impact the ability to mount an effective immune response to infection and/or vaccination. Recently reported findings indicate that SARS-CoV-2-specific IgG antibody dynamics may differ between SARS-CoV-2- infected cancer patients and healthy individuals. Specifically, low seroconversion rates have been reported in patients with hematological malignancies (82%), and in those receiving anti-CD-20 antibody therapy (59%), and stem cell transplantation (60%). By contrast, all patients treated with immunotherapy and particularly immune checkpoint blockade seroconverted (3). Finally, scattered evidence exists that induction chemotherapy causes neither loss of antibody titer nor reactivation of the infection (4, 5).

## Immune Responses Of Fragile Patients: Current Evidence

Main results from current available studies evaluating the immune response in fragile subjects, represented by humoral response in most of the cases, are summarized in **Table 1**. Overall, anti-SARS-CoV-2 antibody response rates in patients diagnosed with malignancies and/or disease requiring immunosuppressive therapies, was considerably low. Specifically, SARS-CoV-2 antibody responses at week 3 following the first dose of the Pfizer BioNTech vaccine were only 39% and 13% in patients with solid and hematological malignancies respectively, compared with 97% in those without cancer (6). After the second dose, the immune response improved significantly for solid tumor patients, with 95% of them showing detectable antibodies to the SARS-CoV-2. By contrast, those who did not get a vaccine boost at three weeks did not display any real improvement, with only 43% of solid tumor patients and 8% of hematologic cancer patients developing antibodies. The poor response to one single dose of vaccine was also confirmed in patients with multiple myeloma and in solid transplant recipients (7, 8). Finally, although the guidance for patients receiving chemotherapy and needing vaccines has been relatively clear so far, it is not yet so for anti-COVID-19 vaccination. In fact, an initial report indicated that vaccine non-responders were also somewhat more frequent among those who received vaccination within 15 days of cancer treatment, particularly chemotherapy (6). This finding needs to be corroborated in order to optimize the timing of vaccination while preserving the anti-cancer treatment schedule. Questions regarding the interference of treatment and disease in seroconversion and anti-SARS-CoV2 vaccine efficacy extend to several other conditions not only related to cancer (6, 7, 9, 10), but including also immunological disorders (8, 9) and disease requiring immunosuppressive therapies (8, 13–15). It is therefore paramount, particularly in the current pandemic situation, to identify a methodology to address key issues about immunogenicity (e.g. the kinetic of immune response and its persistence, the neutralization capability of specific antibodies, the coordination of both humoral and cell-mediated immune responses) to optimize monitoring protocols in the highly complex fragile patient populations. It is also fundamental to evaluate whether some fragile conditions do not allow proper vaccination and these patients should therefore wait to be vaccinated or be protected differently as for instance by creating a ring of protected relations, or should receive a third boosting dose. It is also imperative to assess whether the vaccination schedule should take into account patient treatments and be delayed to avoid interference. In a time of vaccine scarcity, these issues are important for a proper vaccination national plan. A comprehensive study of the immune response in fragile patients will allow in the future to take informed decisions on the questions raised above.

**Table 1:** Immune responses of fragile patients: current evidence.

Author	Disease	Vaccines	n	Immune assessment	Timing of assessment		Vaccine responders	Comments
					1^ dose (n)	2^ dose (n)		
Monin-Aldama L, 2021 (5)	Solid and haematological malignancies	BNT162b2	151	Antibody seroconversion, T cell responses, and neutralisation of SARS-CoV-2 Wuhan strain and of a variant of concern (B.1.1.7)	X (118)	X (30)	15%-40%	Efficacy increased by boosting at 21-days (95% within 2 weeks of boost) in solid cancer patients .
Bird S, 2021 (6)	Multiple Myeloma	BNT162b2	93	SARS-CoV-2 IgG antibodies directed against the Spike-protein	X (93)		70%	
		ChAdOx1 nCoV-19						
Boyarsky BJ, 2021 (7)	Organ transplant	BNT162b2mRNA-1273	436	SARS-CoV-2 IgG antibodies directed against the Spike-protein	X (427)		31% (BNT162b2)	Increased antibody response in young, in absence of immunosuppressive therapy, and with mRNA-1273.
							69% (mRNA-1273)	
Herishanu Y, 2021 (8)	CLL	BNT162b2	167	SARS-CoV-2 IgG antibodies directed against the Spike-protein		X (167)	39.5%	Vaccine responders: 79.2% among complete disease responders, 55.2% in treatment-naïve, and 16% in patients under treatment.
Agha M, 2021 (9)	Haematological malignancies	BNT162b2	67	SARS-CoV-2 Ig Ma and IgG antibodies directed against the Spike-protein		X (67)	54%	
		mRNA-1273						
Wong SI, 2021 (10)	Inflammatory bowel disease	BNT162b2	48	SARS-CoV-2 IgG antibodies directed against the Spike-protein, and Nucleocapsid-protein	X (22)	X (26)	71.4 and 100% after the 1^ and 2^ dose	Decreased antibody titers in patients receiving anti-TNF therapies.
		mRNA-1273						
Geisen UM, 2021 (11)	CID	BNT162b2	26	SARS-CoV-2 IgG antibodies directed against the Spike-protein; neutralising antibodies ELISA-based neutralisation test system; SARS-CoV-2 IgA antibodies		X (26)	100%	IgG titres lower in patients with CID as compared with healthy controls. No patients experienced flare up of CID.
		mRNA-1273						
Rabinowich L, 2021 (12)	Organ transplant	BNT162b2	80	SARS-CoV-2 IgG antibodies directed against the Spike-protein, and Nucleocapsid-protein		X (80)	47.5%	
Grupper A, 2021 (13)	Organ transplant	BNT162b2	136	SARS-CoV-2 IgG antibodies directed against the Spike-protein		X (136)	37.5%	
Chavarot N, 2021 (14)	Organ transplant	BNT162b2	101	SARS-CoV-2 IgG antibodies directed against the Spike-protein	X (101)	X (35)	2% and 5.7% after the 1^ and 2^ dose	
		mRNA-1273						

CLL, Chronic lymphocytic leukemia; CID, Chronic inflammatory diseases.

## Challenges

One of the problems in designing a study on fragile subjects is that the in-scope population is very dis-homogeneous and running double blind randomized studies would pose at least two challenges. One challenge would be the number of stratification factors and the sample size required for a study sufficiently powered to provide conclusive results. The second, but not less relevant, challenge would be ethical as in the presence of an approved vaccine it would be difficult to offer only placebo to a subject who could potentially benefit of an intervention. Given the difficulties in studying these specific situations it is quite understandable why, thus far, no MAH (marketing authorization holder) has initiated any specific study and has left to the health authorities/treating physician the final benefit/risk recommendation for the single individual.

To better understand the type of immunologic response elicited by the vaccination in selected subgroups it is important to define the different categories to be studied. Firstly, some immunologic conditions are *per se* criteria for an altered immunocompetence such as for instance the clinical situation of patients receiving organ or bone marrow transplants and thus needing immunosuppressive therapy. Secondly, patients with a diagnosis of cancer are another subgroup which for different reasons may have alterations of their immune response and which can be further segmented in patients with advanced solid tumors (recurrent, metastatic, extended solid tumors), patients with a diagnosis of cancer (solid tumors) not necessarily advanced but under treatment (for instance chemotherapy but also antibody drug conjugates and or either immune therapies and or cell therapies) and patients with hematologic malignancies.

## Objectives

**Figure 1:**
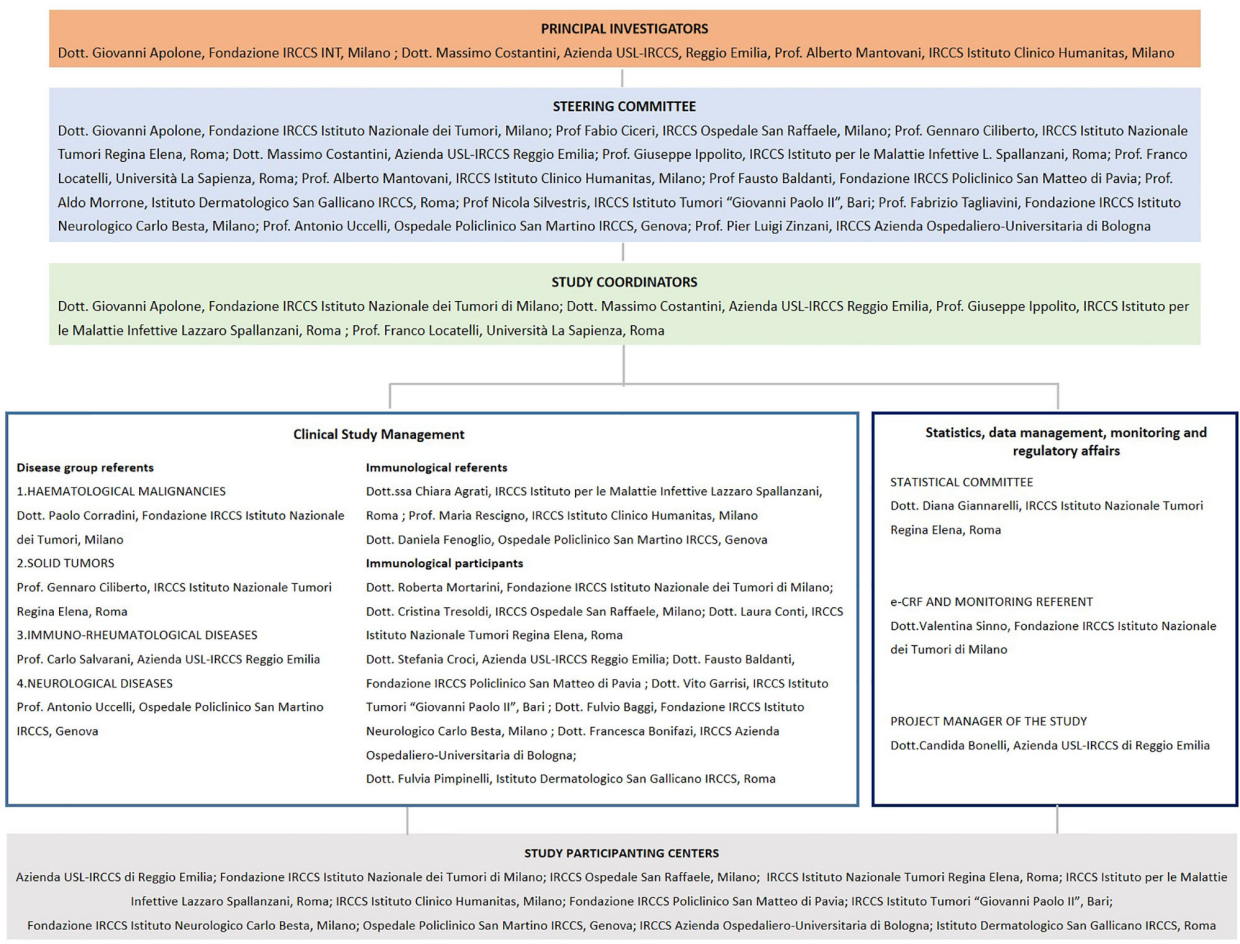
Organogram of the clinical study.

In the context of the national vaccination campaign in which available vaccines are administered to fragile patients, this study would like to assess prospectively the effectiveness of the COVID-19 vaccination in these specific subgroups, and to characterize the kinetics of the immune response to the vaccination and its persistence overtime. Here we have gathered together 12 healthcare centers recognized as centers of excellence by the Italian Ministry of Health (Istituti di Ricovero e Cura a Carattere Scientifico, IRCCS) to share one common protocol of vaccine assessment that has centralized the testing of antibody responses, and their neutralizing activities, and has shared common reagents and technologies for the evaluation of the T cell response to the Spike vaccine protein (**Figure 1**). Hopefully, the characterization of the subgroup responses will allow to shade some light on the true efficacy of the COVID-19 vaccination in the so called altered immunocompetent population.

## Study Design

The VAX4FRIAL study is an observational, multicentre, prospective study with the general objective is to assess the effectiveness of the COVID-19 vaccination in terms of induction of humoral and cell-mediated immune responses in selected fragile (altered immunocompetence) populations, due to their underlying conditions or therapeutic treatments. It will be conducted through the creation of a network between 12 IRCCS in Italy. The study will be carried out in compliance with the Helsinki Declaration. The protocol has been approved by national competent authorities (AIFA) and the ethics committee of National Institute for Infectious diseases Lazzaro Spallanzani (IRCCS), and has been registered in ClinicalTrials.gov (NCT04848493). All patients will sign written informed consent before being enrolled for the study. We also recommended that COVID-19 vaccination studies activated independently by each institution shared the same outline as VAX4FRAIL. This recommendation was made to allow for the possible pooling of data from eligible cases not originally included in the VAX4FRAIL study.

## Participants

The study design plans to recruit 1300 fragile patients, divided among the following subgroups: 1) patients affected by hematological malignancies (n= 500); 2) patients affected by solid tumors (n= 400; 3) patients affected by immunorheumatological diseases, as ANCA-associated vasculitis and interstitial lung disease in autoimmune conditions (n=200 patients); 4) patients affected by neurological diseases, as multiple sclerosis and generalized Myasthenia Gravis (n=200 patients).

## Inclusion Criteria

i) Age ≥ 18 years, ii) undergoing COVID-19 vaccination with Pfizer-BioNTech or Moderna vaccine; iii) life expectancy of at least 12 months at the time of vaccine administration, according to the clinician’s evaluation; iv) Ability to understand and the willingness to sign a written informed consent document; v) Signed Informed Consent Form.

## Exclusion Criteria

i) Patients with psychiatric illness/social situations that would limit compliance with study requirements; ii) History of laboratory-confirmed SARS-CoV-2 infection.

A hemogram will be performed for each patient before vaccination to evaluate the baseline levels of neutrophils, lymphocytes and monocytes. A control group of subjects without evidence of specific disease will consist of 300 Health Care Providers collected by IRCCS Spallanzani, Rome, receiving the same vaccination treatments, and assessed for humoral and cell-mediated immune responses with the same procedures of this protocol.

## Procedures

**Figure 2:**
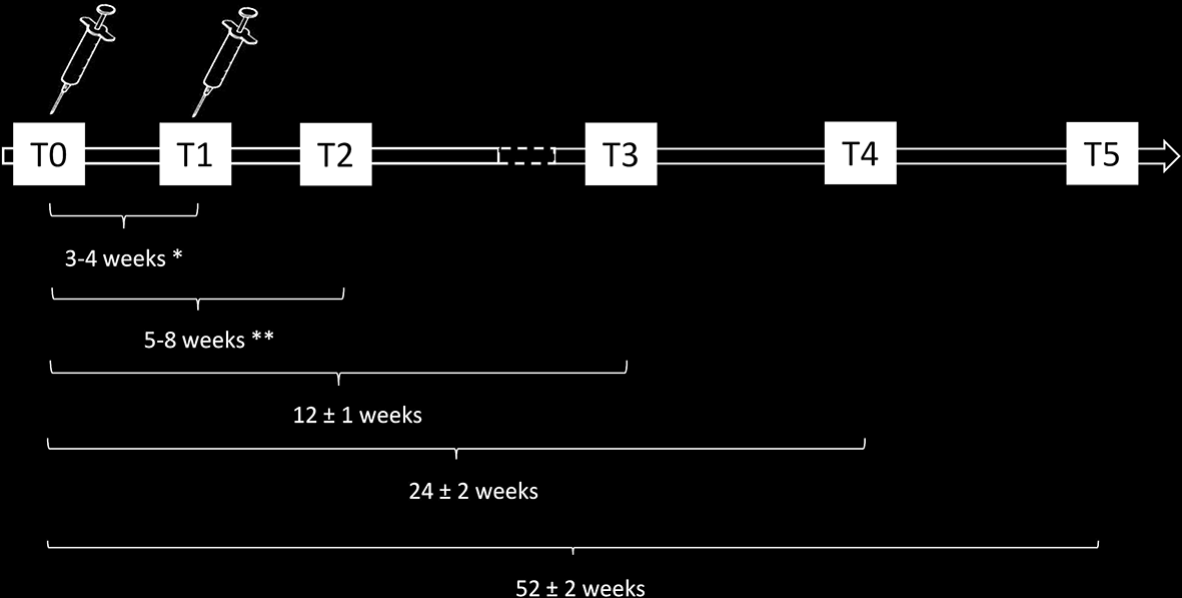
Schematic representation of the timeline of immunomonitoring of the clinical study.

According to the national and regional indications, patients enrolled in this study will be vaccinated with either the Pfizer/BioNTech or with Moderna vaccines. Patients will be monitored for antibodies and cellular immunity directed against the SARS-CoV-2 spike antigen for 12 months or until death. In particular, blood samples for immunological testing will be performed at 6 time points (**Figure 2**) as follows: T0, the day of vaccination; T1, the day of the booster dose (according to the schedule of the vaccines: 3 weeks after T0 for patients receiving Pfizer/BioNTech vaccine, 4 weeks after T0 for patients receiving Moderna vaccine); T2, between 5 and 7 weeks after T0 for patients receiving Pfizer/BioNTech vaccine and between 6 and 8 weeks after T0 for patients receiving Moderna vaccine; T3, 12 (± 1) weeks after T0; T4, 24 (± 2) weeks after T0; T5, 52 (± 2) weeks after T0.

## Immunological Efficacy Assessment

Humoral response: For each time point the humoral immune response to vaccination will be assessed by a serological assay able to detect anti-Spike IgG antibodies in sera Possible natural infection before or after vaccination will be assessed by measuring anti-Nucleocapsid IgG antibodies. Patients with potential concurrent SARS-CoV-2 infection identified through anti-N increased level at the same time point will be excluded by this analysis. On a subset of subjects, at 2-4 weeks after the booster dose and at 12, 24 weeks after first vaccine administration, a neutralization assay will be performed to evaluate the functional activity of vaccination induced anti-Spike antibodies. Briefly, sera from patients will be tested for their ability to neutralize SARS-CoV-2 infection of VERO-E6 cells in a BSL-3 facility.

T cell immunity: The cell-mediated immune response to vaccination will be assessed by a first-line test standardized on whole blood assay. Briefly, at the laboratory of each clinical center heparin blood samples will be stimulated in the absence/presence of a pool of peptides spanning the S protein (S: PepTivator^®^ SARS-CoV-2 ProtS, PepTivator^®^ SARS-CoV-2 ProtS1; PepTivator^®^ SARS-CoV-2 ProtS+, purchased by Miltenyi Biotech) for 20 hours at 37°C 5%CO2. The day after, conditioned plasma will be harvested and stored to quantify the release of IFNγ, TNFα, IL-2 and IP-10 cytokines by automated Immunoassay assay (Protein Simle, Biotechne). Moreover, peripheral blood mononuclear cells (PBMC) will be isolated and stored to allow subsequent other T cell assay (e.g., ELISpot assay).

All serological assays, quantification of released cytokines in conditioned plasma and ELISpot assay will be centralized in the Spallanzani laboratory to generate homogeneous data by sharing reagents and technologies.

## Data Collection

A standard electronic case report form (e-CRF) will be designed at the beginning of our study by the Fondazione IRCCS Istituto Nazionale dei Tumori, Milano Clinical Trial Center. It will be used to obtain demographic information, disease history, surgical details, imaging features and outcome events of participants. Following registration, patient data will be collected in the e-CRF. Clinical and centrally collected and processed laboratory data will be analysed by the study sponsor IRCCS Reggio Emilia.

## Sample Size

In this context of high uncertainty on short- and long-term effect of vaccination, the approach to this observational study will be mainly descriptive and based on confidence intervals rather than hypotheses testing. According to immunological tests on a sample of 250 Health Care Providers analysed in the laboratories of the IRCCS Regina Elena, Rome, the geometric mean titer after one week from the second vaccine administration was about 286 AU/ml with a 95% confidence interval ranging from 250 to 328.

As an indication of how many patients we could include for each subgroup we may consider the use of the Student t-test on logarithm of concentration to assess differences in titer at a specific time-point between subgroups of patients and subjects without evidence of disease.

Under these assumptions a sample size of 100 patients for each subgroup will allow to assess differences in terms of geometric mean of titers of about 80 AU/ml with a power of 80% at a significance level of 5% when the standard deviation, on the logarithmic scale, is equal to 1.

## Statistical Analysis

Descriptive analyses will be carried out and include statistics such as arithmetic mean, median and geometric mean, with corresponding 95% CIs for continuous outcomes generated by the immunological tests at each time point (at first dose, at second dose, 2 weeks after the second dose, at 3, 6 and 12 months after first vaccine administration). Percentages with corresponding 95% CIs will be computed to describe the frequency of COVID-19 infection and to report adverse events.

Analyses at specific time-points will be considered as well as a repeated measurements ANOVA will be implemented to study the trend over time in each subgroup with respect to subjects without evidence of considered diseases.

Analyses will be carried out overall and within each subgroup (underlying conditions and referents) to point out differences related to the specific condition. Sub-group analyses will be performed also for socio-demographic subgroups and comorbidities according to the consistency and other relevant variables of each subgroup. No missing data imputations is planned.

## Expected Outcomes

We consider the VAX4FRAIL study to be important for the Italian Health Care System not only because of the unquestionable value of the evidence that will produce in the national and international context, which will be able to guide the clinical and healthcare behavior of these citizens, whose numbers are estimated at many millions, but also and above all because it will represent a model of collaboration between Research Institutes that will be useful in the future to answer questions that are relevant for scientific and policy priorities. As we will collect metadata of the patients, including treatments, demographical parameters, comorbidities, etc. we will perform post-hoc sub-analyses to correlate the immune response to the collected features. Given the heterogeneity of the patients, we have not included these analyses in the study objectives but statistically relevant, they will be particularly instructive to take informed decisions on which patients will be needing a third dose and the type of treatments to avoid or postpone in order to favor immune induction.

Going beyond considering simply the scientific results of the combined study, there are a number of further advantages, including: the use of standardized and validated diagnostic procedures throughout the country, the possibility of building a cohort to be followed over time, which can also be used to decide on possible recalls or for scheduling the use of monoclonal antibodies for high-risk patients (non-responders), the possibility of building a collection of biological samples for future studies (bio-banks).

This initiative confirms the absolute centrality of the IRCCS network, a unique model on the international scene, which proposes itself as the fundamental element of the NHS (16). It is now considered as a national sentinel system for monitoring the response to the vaccination campaign in particularly fragile subjects, and in the future, it will be the most appropriate system for responding to other health issues in order to optimize regional and national health policies.

## Author Contributions

CA, NS, SDC, GA, AMa contributed to conception and design of the study. SC, DF organized the database. CA, SDC, MC, DG, GI wrote the first draft of the manuscript. All authors contributed to the article and approved the submitted version.

## Conflict of Interest

The authors declare that the research was conducted in the absence of any commercial or financial relationships that could be construed as a potential conflict of interest.

The reviewer AT declared a shared affiliation with one of the authors, FC, to the handling editor at the time of review.

## Publisher’s Note

All claims expressed in this article are solely those of the authors and do not necessarily represent those of their affiliated organizations, or those of the publisher, the editors and the reviewers. Any product that may be evaluated in this article, or claim that may be made by its manufacturer, is not guaranteed or endorsed by the publisher.
